# Technological tools for assessing children's food intake: a scoping review

**DOI:** 10.1017/jns.2023.27

**Published:** 2023-04-11

**Authors:** Jonas de Souza Mata, Jade Veloso Freitas, Sandra Patricia Crispim, Gabriela S. Interlenghi, Marcela Baraúna Magno, Daniele Masterson Tavares Pereira Ferreira, Marina Campos Araujo

**Affiliations:** 1 Emília de Jesus Ferreiro School of Nutrition, Federal Fluminense University, Niterói, RJ, Brazil; 2 Department of Epidemiology, Institute of Social Medicine, Rio de Janeiro State University, Rua São Francisco Xavier, 524, 7° andar, bloco E, sala 6004, Maracanã, Rio de Janeiro, RJ CEP 20550-900, Brazil; 3 Department of Nutrition, Federal University of Paraná, Curitiba, PR, Brazil; 4 Independent Researcher, Rio de Janeiro, RJ, Brazil; 5 Associate Professor of Graduate Studies in Dentistry, Veiga de Almeida University, Rio de Janeiro, RJ, Brazil; 6 Health Sciences Center, Central Library, Federal University of Rio de Janeiro, Rio de Janeiro, RJ, Brazil; 7 Sérgio Arouca National School of Public Health, Oswaldo Cruz Foundation, Ministry of Health, Rio de Janeiro, RJ, Brazil

**Keywords:** Children, Dietary assessment, Epidemiology, Food intake, Technology

## Abstract

Technological innovations can standardise and minimise reporting errors in dietary assessment. This scoping review aimed to summarise the characteristics of technological tools used to assess children's food intake. The review followed the Joanna Briggs Institute's manual. The main inclusion criterion was studied that assessed the dietary intake of children 0–9 years of age using technology. We also considered articles on validation and calibration of technologies. We retrieved 15 119 studies and 279 articles were read in full, after which we selected 93 works that met the eligibility criteria. Forty-six technologies were identified, 37 % of which had been developed in Europe and 32⋅6 % in North America; 65⋅2 % were self-administered; 27 % were used exclusively at home; 37 % involved web-based software and more than 80 % were in children over 6 years of age. 24HR was the most widely used traditional method in the technologies (56⋅5 %), and 47⋅8 % of the tools were validated. The review summarised helpful information for studies on using existing tools or that intend to develop or validate tools with various innovations. It focused on places with a shortage of such technologies.

## Introduction

Conventional methods for dietary assessment emerged in the 20th century, and the first written records date back to the 1930s in the United States and European countries, especially in the United Kingdom. With the increase in dietary records, technological progress has facilitated computer access since the 1970s and allowed nutritional calculations through dietary assessment incorporated into computerised systems^(1)^. Innovations that retrieve dietary data from the population were established, including software with online and offline functionality, personal digital assistants (PDAs), web-based technologies (WBTs), apps for mobile data collection devices, barcode readers, digital cameras and sensors coupled to clothing^(2)^.

Despite all this technological progress, many tools are still based on traditional methods that consider self-reported food intake, such as 24-hour recall (24HR), food records (FR) and the food frequency questionnaire (FFQ)^(3,4)^. Thus, inherent errors in the traditional dietary assessment are also found in the technologies, such as underestimating food intake^(5)^. Meanwhile, increasingly more technological innovations that operate independently of conventional methods and self-report, such as barcode readers, technologies based on digital cameras, and sensors are emerging^(4)^. Even so, all technological tools are subject to measurement and estimation errors, and validation studies increasingly aim to quantify such errors^(6)^.

Especially in childhood dietary assessment, these errors may be magnified by the child's cognitive capacity limitation, requiring a parent's assistance in reporting the diet^(6)^ and potentially expanding the possibility of food intake estimation errors, such as under- or overreporting^(7)^. However, the investigation of children's dietary intake is essential in assessing their nutritional status and predicting their health status in subsequent life stages. Eating habits established in the early decades of life are known to significantly affect the risk of developing chronic diseases, especially overweight and obesity, in childhood and at future ages^(8)^. Studies have thus developed technologies that minimise errors in childhood dietary assessment and automate and standardise data collection, integrating technological and digital resources that facilitate food measurement, reducing costs and increasing individuals’ participation rates, facilitating data collection^(7)^.

This context underscores the relevance of knowing the existing technological tools for assessing children's food intake, including the tools’ characteristics and validity. Such knowledge is essential for assisting the choice of available tools, verifying populations in which these technological resources are still scarce, and identifying trends and possibilities for improving and developing innovations applied to dietary assessment technologies. Thus far, seven reviews have shown technologies for obtaining data on children's food intake^(2,6,7,9–12)^. Still, some reviews failed to follow standard methodologies for reviews based on international guidelines^(9,12)^, addressed different age groups, not specific to children^(2,6,7,9–11)^, and only retrieved validation studies^(7,10)^, or were limited to one type of dietary assessment method^(6,12)^. Therefore, the principal question in the present study was the following: Which technologies are used to assess children's food intake? A scoping review was used to answer this question to identify and characterise the technological tools used to assess children's food intake.

## Methods

### Study design

A scoping review was conducted per the *Joanna Briggs Institute Reviewer Manual*^(13)^ and with additional methodological guidelines^(14)^. The review followed the PRISMA-ScR verification list (*Preferred Reporting Items for Systematic Reviews and Meta-Analyses Extension for Scoping Reviews*) (Supplementary Table S1)^(15)^. The protocol was registered with the *Open Science Framework* (https://doi.org/10.17605/OSF.IO/WMBFZ)^(16)^ on 14 April 2021. The principal question that gave rise to the search strategy was formulated with the mnemonic PCC (Population, Concept and Context)^(13)^, where the population was defined as children 0–9 years of age, the concept as technological tools and context as food intake assessment.

### Eligibility criteria

The eligibility criteria were established *a priori* (Supplementary Table S2). Studies were eligible if they assessed the food intake of children 0–9 years of age using technology. Studies that assessed other age groups and included children 0–9 years were included. Articles on validation and calibration of technological tools were also considered. Studies with objectives other than assessment of children's food intake but which at some stage performed and described the technology used were also included. The review excluded studies that exclusively used traditional dietary assessment tools or technologies that were only used in the data analysis or for some purposes other than assessing food intake, such as dietary education, food preferences, promotion of healthy eating habits (mainly clinical trials), body weight control and exclusive assessment of breastfeeding. The review also excluded review studies, protocols, abstracts and posters published in congress proceedings, articles written in languages other than Portuguese, English and Spanish, and studies that did not present sufficient information for data extraction.

### Search strategy

The systematized search process was conducted in five databases and oriented by a librarian with experience in synthesis studies (DM). The first database explored was MEDLINE (Medical Literature Analysis and Retrieval System Online) via PubMed. The strategy was subsequently customised for the databases Scopus, Web of Science, LILACS (Latin American and Caribbean Literature in Health Sciences) via BVS and the Cochrane Library. The search was performed up to 26 October 2021, in all the databases. A complementary search was performed in the gray literature and explored in the *OpenGrey* source. Studies were also identified by cross-referencing selected relevant studies in contact with authors. No restrictions were imposed on the date of publication or languages. The complete search strategy is shown in Supplementary Table S3.

### Article selection and data extraction

#### Initial selection (Reading titles and abstracts)

All the identified references were organised as a dataset in the Endnote software, version x7 for reading the titles and abstracts, and duplicates were removed.

Three researchers read the studies’ titles and abstracts individually (GSI, JSM and MBM), and the article selection followed the eligibility criteria. The study coordinator, a fourth member (MCA), evaluated any disagreements in the article selection process.

#### Final selection (Reading full texts and data extraction)

The potentially relevant studies in the first stage were retrieved for reading the full texts via PubMed (https://pubmed.ncbi.nlm.nih.gov/) and *Google Scholar* (https://scholar.google.com.br/) websites. Some articles that remained inaccessible were requested from the authors via e-mail, but this method was unsuccessful. Full-text reading was performed pairwise, but two researchers joined one of the pairs and shared the reading (GSI and JSM + GF). Divergences in the articles were resolved by another researcher (MCA).

The extraction table created to compile the critical information on the technological tools retrieved the following data: characteristics of the technological tools (brand name; type of technology; food intake assessment method; administration method; data entry method; age group; language displayed by the software; country of origin; data collection environment and references), details on the technologies (number of foods/preparations/beverages available in the database; search format/food insertion in the technology; assessing amounts consumed; method for estimating portions; nutrient intake assessment; supplement consumption report; person reporting the child's food intake; food composition database) and technology validation (reference method; the number of participants; statistical analysis used; participants’ characteristics – age group, sex and study location; and principal results and conclusions).

### Data analysis and synthesis

A qualitative synthesis of the selected studies was used to map the literature, as designed in the research question. Absolute and relative frequencies were calculated to synthesise some information, using Microsoft Excel and R (version 4.2.0) to analyse the data.

## Results

**Fig. 1.**
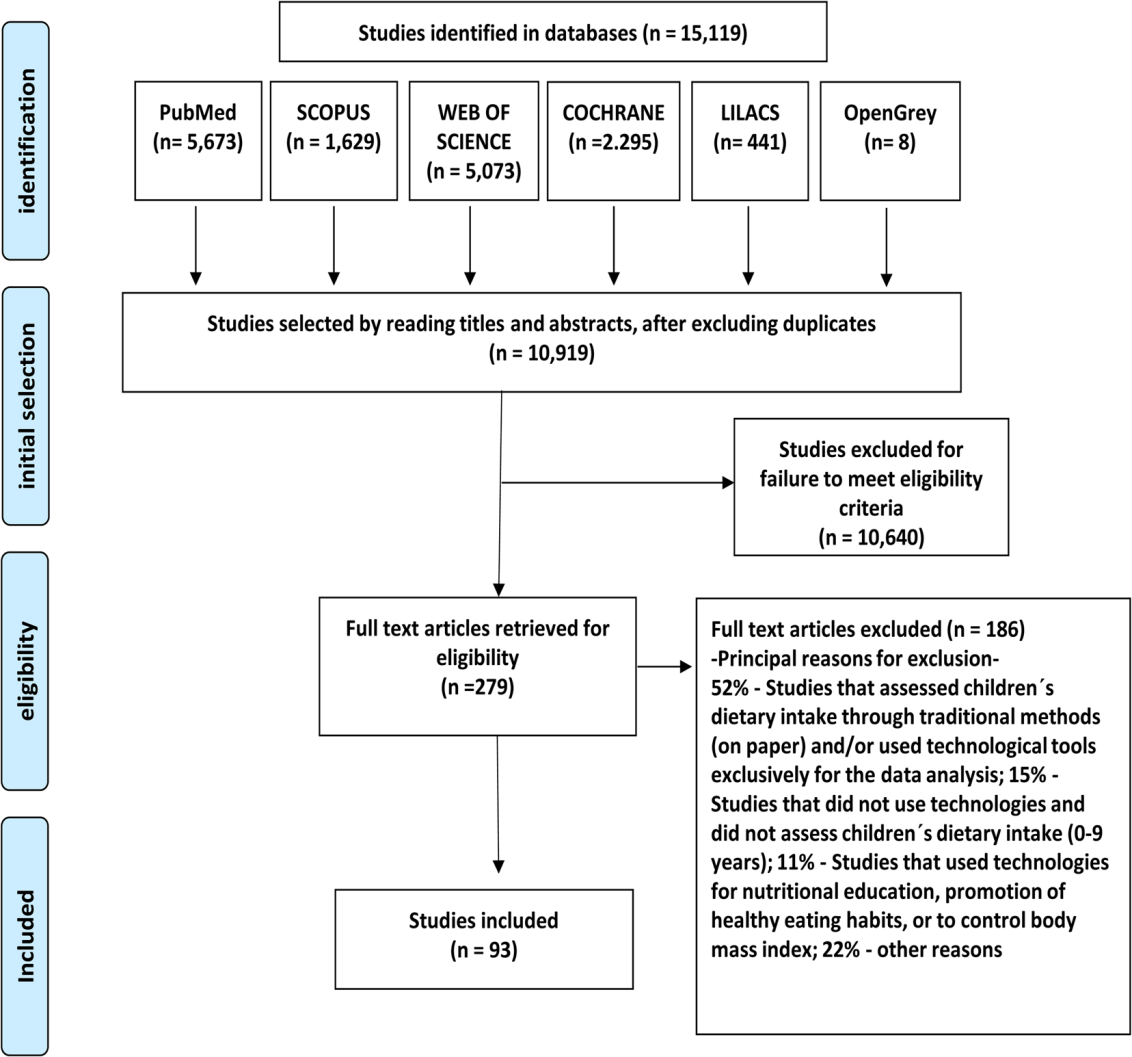
Flowchart describing the scoping review process.

A total of 15 119 studies were retrieved from the databases, yielding 10 919 articles after excluding duplicates. Then, titles and abstracts were read, and the inclusion and exclusion criteria were applied, resulting in 279 articles eligible for full-text reading. Ninety-three studies finally met the eligibility criteria (Fig. 1). The principal reasons for exclusion were the use of traditional dietary assessment methods or technology exclusively for data analysis (*n* 96) and technology applied to nutritional education, promotion of healthy habits, body weight control and analysis of eating behaviours or dietary preferences (*n* 20) (Supplementary Table S4).

A total of forty-six technologies were identified in the ninety-three studies analysed, in which the most widely studied technologies were Web-DASC, addressed by nine articles (9⋅7 %)^(17–25)^, Nutrition Data System for Research (NDSR), cited in seven articles (7⋅5 %)^(26–32)^, and EPIC-Soft^(39–43)^ and Web-CAAFE^(44–48)^, each mentioned in five articles (5⋅7 %). Most of the technologies were developed in Europe (37 %)^(17–25,33,39–43,49–72)^, North America (32⋅6 %)^(26–32,73–94)^ and Oceania (10⋅9 %)^(38,95–98)^. The most widely used traditional dietary assessment method used in the tools was 24HR (56⋅5 %; *n* 26)^(17–37,39–48,52,60,61,77–81,85,86,89,90,94–97,99–107)^. Thirty technologies (65⋅2 %) were self-administered^(17–25,33–38,44–52,54–61,65,67,68,70–84,86–88,91,94,100,102–105,107–110)^, while 11 (23⋅9 %) were administered by an interviewer^(26–32,39–43,69,85,89,90,95,97–99,101,106)^ and 4 (10⋅9 %) had some other form of administration or did not provide this information^(53,62–64,66,92,93)^. The primary data collection settings were home and school. Approximately 27 % of the technologies were used exclusively at home^(17–32,38–43,54–57,65,67,68,71,84,85,87–91,98,99,101,106,107)^, 22⋅6 % at school^(33–37,44–52,60–64,66–69,77–80,91,94,95,100,102–105)^ and 6⋅5 % in both settings^(58,59,82,83,92,93,96,97,108–110)^. The leading types of technology were web-based software programs (37 %; *n* 17)^(17–25,33–37,44–52,58,59,67,68,80,81,84,86,87,94,95,100,108)^ and offline programs (30⋅4 %; *n* 14)^(26–32,39–43,53,60,61,65,69,77–79,85,88–90,96,97,99,101–105)^. Digital cameras were only used in 11 % of the technologies (*n* 6)^(54–57,71,82,83,91–93,109,110)^. More than 80 % of the studies were conducted in the age group over 6 years^(17–25,30,33–52,60–70,73,74,77–80,82–91,93,95–106,108–110)^ (Table 1).

**Table 1.** Basic characteristics of technologies used to assess children's dietary intake

Brand name (tool used)	Type of technology	Food intake assessment method	Method of administration	Data entry method	Age group	Language displayed by the software	Country of origin	Data collection setting	References
*Web-based Food Record* (WebFR)	Web-based software	FR with 24HR's elements	Self-administered	Text	8–9 years (Medin *et al.*, 2015) 8–9 and 12–14 years (Medin *et al.*, 2016, 2017)	Norwegian	Norway	School	Medin *et al.*^(49–51)^
Zambia Tablet-based 24 h recall Tool	Software with offline functionality	24HR	Interview	Text	4–8 years	English	Zambia	Home	Caswell *et al.*^(99^)
*Portuguese self-administered* *computerised 24-hour* *dietar*y (PAC-24)	Web-based software	24HR	Self-administered	Text	7–10 years	Portuguese	Portugal	School	Carvalho *et al.*^(52^)
*Mobile Food Record* (mFR)	Smartphone App	Analysis of images by FR	Self-administered	Food Photos	3–10 years (Aflague *et al.*, 2015) 8–18 years (Polfuss *et al.*, 2018) 3–12 months (Campbell *et al.*, 2020; Fialkowski *et al.*, 2020)	English	USA	Not applicable	Aflague *et al.*; Polfuss *et al.*; Campbell *et al.*; Fialkowski *et al.*^(73–76)^
*Dietetyk2 Nutritional Program*	Software with offline functionality	Not informed	Not reported	Not reported	3–6 years	Not reported	Poland	Not reported	Ambroszkiewicz *et al.*^(53)^
*Web-based Dietary Assessment Software for Children* (WebDASC)	Web-based software	24HR	Self-administered	Text	8–11 years (All the studies)	Danish	Denmark	Home	Andersen *et al.*; Biltoft-Jensen *et al.*; Kjeldsen *et al.*^(17–25)^
*Food Intake Recording Software System* (FIRSSt)	Software with offline functionality	24HR	Self-administered	Options for answers	9–11 years (Baranowski *et al.*, 2002) 8–12 years (Baranowski *et al.*, 2003) 9–10 years (Cullen *et al.*, 2004)	English	USA	School	Baranowski *et al.*; Cullen *et al.*^(77–79)^
*Automated Self-Administered 24 Hour Dietary Recall* (ASA24)	Web-based software	24HR	Self-administered	Not reported	8–13 years	English	USA	School	Baranowski *et al.*^(80)^
*Food Intake Recording Software System version 4* (FIRSSt4)	Web-based software	24HR	Self-administered	Options for answers	Not informed	English	USA	Not reported	Baranowski *et al.*^(81)^
*eButton*	Digital camera	Analysis of images	Self-administered	Food Photos	9–13 years (Beltran *et al.*, 2018) 8–13 years (Beltran *et al.*, 2016)	English	USA	Home and school	Beltran *et al.*^(82,83)^
*Nutrition Data System for Research* (NDSR)	Software with offline functionality	24HR	Interview	Text	12 months (Bonuck *et al.*, 2014) 4–24 months (Briefel *et al.*, 2006; Devaney *et al.*, 2004; Ponza *et al.*, 2004) 0–47 months (Butte *et al.*, 2010) 8–10 years (Lanctot *et al.*, 2008) 9–11 years (Van Furth *et al.*, 2016)	English	USA	Home – by telephone – (Bonuck *et al.*, 2013)	Bonuck *et al.* Briefel *et al.*; Butte *et al.*; Devaney *et al.*; Lanctot *et al.*; Ponza *et al.*; Thompson *et al.*^(26–32)^
*RealityMalta*	Web-based software	24HR	Self-administered	Not reported	9–11 years	English	Malta	School	Copperstone *et al.*^(33)^
*Consumo Alimentar e Atividade Física de Escolares* (CAAFE)	Web-based software	24HR	Self-administered	Options for answers with figures	6–12 years (da Costa *et al.*, 2013a, 2013b) 7–10 years (Davies *et al.*, 2015a) 7–11 years (Davies *et al.*, 2015b)	Portuguese	Brazil	School	da Costa *et al.*; Davies *et al.*^(34–37)^
*Evernote app*	Smartphone App	FFQ	Self-administered	Pictures of foods and text	9–12 years	English	New Zealand	Home	Davison *et al.*^(38)^
*European Prospective Investigation into Cancer and Nutrition Software* (EPIC-soft)	Software with offline functionality	24HR	Interview	Pictures of foods and text	7–8 years and 12–13 years (de Boer *et al.*, 2011a, 2011b; Trolle *et al.*, 2011a, 2011b-1) 4–5 years (Trolle *et al.*, 2011b-2)	Not reported	Denmark and Basque region (Spain)	Home	de Boer *et al.*; Trolle *et al.*^(39–43)^
*Consumo Alimentar e Atividade Física de Escolares* (Web-CAAFE)	Web-based software	24HR	Self-administered	Options for answers with figures of foods	7–11 years (Kupek *et al.*, 2016b; Perazi *et al.*, 2020) 7–15 years (Jesus *et al.*, 2017) 7–12 years (Pereira *et al.*, 2020) 7–10 years (Segura *et al.*, 2019)	Portuguese	Brazil	School	Kupek *et al.*; de Jesus *et al.*; Perazi *et al.*; Pereira *et al.*; Segura *et al.*^(44–48)^
*The VioScreen FFQ*	Web-based software	FFQ	Self-administered	Options for answers with figures of foods	6–14 years	English	USA	Home	Deierlein *et al.*^(84)^
*Tool for Energy Balance in Children* (TECH)	Digital camera	FR	Self-administered	Food photos and text	5–6 years (Delisle Nyström *et al.*, 2016) 4 years (Cadenas-Sanchez *et al.*, 2017) 3 years (Henriksson *et al.*, 2015) 4 years (Parekh *et al.*, 2018)	Sweden	Sweden	Home	Cadenas-Sanchez *et al.*; Delisle Nystrom *et al.*; Henriksson *et al.*; Parekh *et al.*^(54–57)^
*Nutrient Data System*	Software with offline functionality	24HR	Interview	Not reported	4–10 years	English	USA	Home (by telephone)	Derr *et al.*^(85)^
*Automated Self-Administered 24 Hour Dietary Recall for Children* (ASA24-Kids-2012)	Web-based software	24HR	Self-administered	Not reported	9–11 years	English	USA	Childcare centres	Diep *et al.*^(86)^
*Previous Day Food Questionnaire* (PDFQ)	Web-based software	24HR	Self-administered	Options for answers with pictures of foods	7–12 years	Portuguese	Brazil	School	Engel *et al.*^(100)^
*Intake24 software*	Web-based software	24HR	Interview	Text	8–11 years	English	New Zealand	School	Eyles *et al.*^(95)^
*European Prospective Investigation into Cancer and Nutrition Software (EPIC-soft Data Entry)*	Software with offline functionality	24HR	Interview	Options for answers and text	4 months–10 years	Not reported	Not reported	Home (in person or by telephone)	Freisling *et al.*^(101)^
Self-Administered Children and Infant Nutrition Assessment (SACINA)	Software with offline functionality	24HR	Self-administered	Options for answers	2–9 years (Graffe *et al.*, 2019) 4–10 years (Börnhorst *et al.*, 2013) 2–9 years (Börnhorst *et al.*, 2014; Svensson *et al.*, 2014)	Not reported	Not reported	School	Börnhorst *et al.*; Graffe *et al.*; Svensson *et al.*^(102–105)^
No brand name (Canada)	Web-based software	FFQ	Self-administered	Options for answers	6–12 years	Not reported	Canada	Home	Bischoff and Portella^(87)^
No brand name (Australia)	Computer-based	24HR	Interview	Text	2–16 years	Not reported	Not reported	Home	Clifton *et al.*, 2011^(106)^
Audio-enhanced tablet computer-based food frequency questionnaire (ATC-based FFQ)	Software with offline functionality	FFQ	Self-administered	Text	2–13 years	English and Spanish	USA	Home	Kilanowski *et al.*^(88)^
No brand name (Norway)	Web-based software	FFQ and 24HR	Self-administered	Not reported	3–5 years (Kristiansen *et al.*, 2017, 2019)	English	Norwegian	Home e school	Kristiansen *et al.*^(58,59)^
Young Adolescents’ Nutrition Assessment on Computer (YANA-C)	Software with offline functionality	24HR	Self-administered	Not reported	9–11 years (Lahoz-Garcia and Garcia-Hermoso, 2018; Lahoz-Garcia *et al.*, 2015)	Spanish	Belgium	School	Lahoz-Garcia and Garcia-Hermoso; Lahoz-Garcia *et al.*^(60,61)^
No brand name (United Kingdom)	Smart card system	Not applicable	Barcode reading	Barcode	8–11 years	English	United Kingdom	School (cantina school)	Lambert *et al.*^(62–64)^
United States Department of Agriculture Automated Multiple-Pass Method program (USDA AMPM)	Software with offline functionality	24HR	Interview	Not reported	≥4 years (Lee *et al.*, 2014) 1–13 years (Shakur *et al.*, 2012)	English	USA	Home	Lee *et al.*; Shakur *et al.*^(89,90)^
ENIA-soft	Software with offline functionality	FR and food propensity questionnaire for supplements	Self-administered	Text	6–17 years	Spanish	Spain	Home	López-Sobaler *et al.*^(65)^
No brand name (Poland)	Smart card system	FR	Barcode reading	Barcode	6–12 years	Not applicable	Poland	School	Luszczki *et al.*^(66)^
No brand name (USA)	Digital camera	FR	Self-administered	Food Photos	9–12 years	English	USA	Home	Matthiessen *et al.*, 2011^(91)^
Synchronised Nutrition and Activity Program™ (SNAP™)	Web-based software	24HR and FFQ	Self-administered	Text	7–15 years (Moore *et al.*, 2007, 2013)	English	England	Home	Moore *et al.*^(67,68)^
No brand name (Reino Unido)	Software with offline functionality	24HR and FFQ	Interview	Text	9–11 years	English	England	School	Moore *et al.*^(69)^
No brand name (USA Nick)	Digital camera	Analysis of images	Collected by interviewers	Food photos	3 years and 8 months–5 years (Nicklas *et al.*, 2012) 3 years and 10 months–5 years (Nicklas *et al.*, 2017) 8–11 years (Taylor *et al.*, 2014)	Not reported	USA	Home and school	Nicklas *et al.*; Taylor *et al.*^(92,93)^
Personal Digital Assistants (PDAs)	App	FR	Self-administered	Pictures of foods	9–15 years	Not reported	Spanish	Hospital	Oliver *et al.*^(70)^
No brand name (New Zealand)	Software with offline functionality	24HR	Interview	Options for answers and text	5–14 years (Parnell *et al.*, 2003; Thomson *et al.*, 2007)	Not reported	New Zealand	Home and school	Parnell *et al.*; Thomson *et al.*^(96,97)^
Computer-assisted telephone interview (CATI)	Computer-based	FFQ	Interview	Text	4–12 years	English	Australia	Home	Sanigorski *et al.*^(98)^
Automated Self-Administered 24-Hour Dietary Assessment Tool (ASA24-Canada)	Web-based software	24HR	Self-administered	Text	2–5 years	English	Canada	School	Wallace *et al.*^(94)^
No brand name (Sweden)	Digital camera	FR	Self-administered	Food photos	12 months	Not applicable	Sweden	Home	Johansson *et al.*^(71)^
My E-Diary and Lifestyle (MEDAL)	Web-based software	FR	Self-administered	Options for answers and text	7–13 years	English	Singapore	Home and school	Chia *et al.*^(108)^
Digital photography (no brand name)	Digital camera	FR	Self-administered	Food photos	7–8 years (Erkilic *et al.*, 2020) 5–7 years (Norman*et al.*, 2020)	Not applicable	Not applicable	Home and school	Erkilic *et al.*; Norman *et al.*^(109,110)^
*Multiple-Pass 24 Diet* (MP24Diet)	Smartphone App	24HR	Self-administered	Options for answers	6–23 months	Not reported	Indonesia	Home	Htet *et al.*^(107)^
NutriKids	Smartphone App	FR	Self-administered	Options for answers and recognition of food's brand and specificities (using camera)	Not reported	French	Switzerland	Hospital	Jacques *et al.*^(72)^

FR, Food Records; 24HR, 24-hour dietary recall; App, Application; USA, United States of America; FFQ, Food Frequency Questionnaire.

**Fig. 2.**
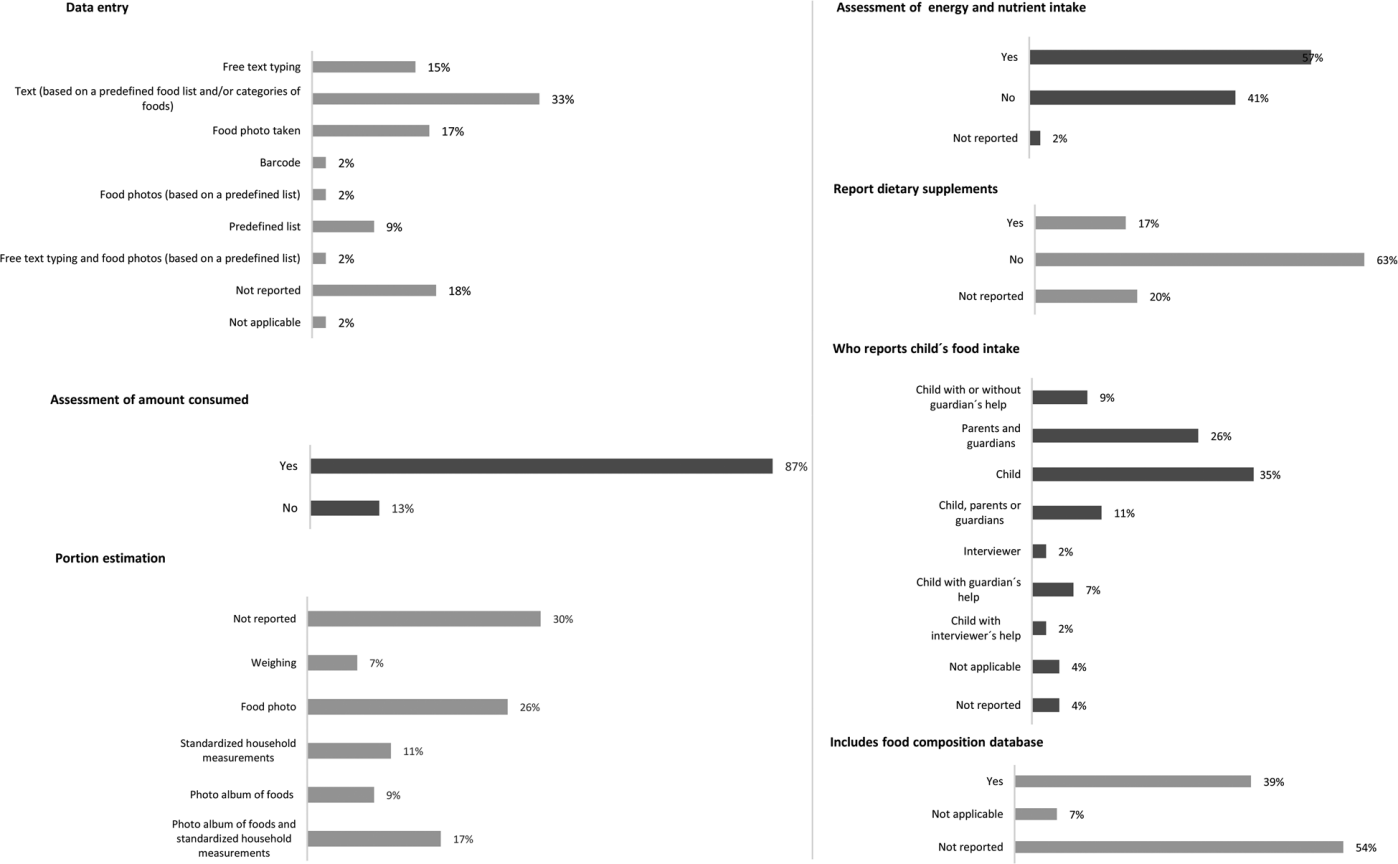
Detailed summary classification of technological tools to assess children's food consumption.

As for the technologies’ details, the principal means for data entry were text format based on a list of names or predefined categories of foods (32⋅6 %)^(34–37,44–48,52,62–64,77–80,87,96–101)^, image capture of foods (17⋅4 %)^(38,54–57,73–76,82,83,91–93)^ and free text (15⋅2 %)^(26–32,49–51,67,68,80,94)^. The data entry method was not informed in 17⋅4 % of the studies^(33,53,60,61,89,90,102–105)^. Most of the technologies were quantitative (87⋅0 %)^(17–33,39–43,49–68,70,71,73–86,88–99,101–105,109,110)^, in which 17⋅4 % of the tools used photo albums and standardised household measures to estimate amounts^(26–32,39–43,49–52,62–65,80,81,84,88,96–99,101–105)^; 8⋅7 % only used photo albums^(17–25,58,59,70,71,85)^ and 26⋅1 % used analysis of food photographs by participants^(54–57,71,73–76,82,83,91–93)^. Most of the technologies allowed estimating energy and nutrient intake (56⋅5 %) ^(39–43,60,61,65,84–87,89,90,92–97,101–105,109,110)^, but only 17⋅4 % described the assessment of food supplement consumption^(65,89,90,96,97,101,109,110)^. Approximately 54⋅6 % of the articles did not report whether the technological tool presented a food composition database^(17–32,39–43,49–52,54–57,60,61,65,67,68,80,82,83,87,89–91,94,96,97,99)^. Children were the informants in approximately 35 % of the tools^(33–38,44–48,52,62–64,70,73–84,86,95,100)^, against 26⋅1 % for parents and guardians^(26–32,54–59,65,71,87,88,94,98,99)^ (Fig. 2).

A total of 47⋅8 % (*n* 22) of the technologies analysed were validated. The tools with most validation studies were Web-DASC with four articles^(21–24)^, followed by Web-FR^(49–51)^ and Web-CAAFE^(37,44,45)^ with three validation studies each. The most widely used reference method for comparing with the technologies in the validation studies was direct observation (38⋅7 %)^(23,37,44,45,49,52,62,77,86,93,94,100)^, followed by 24HR (25⋅8 %)^(33,55,67,77,80,91,109,110)^. The number of participants analysed in the validation studies ranged from 21 to 834. Eighteen studies (58 %) concluded that the validation studies were satisfactory^(21–24,33,38,44,45,50,55,62,67,71,77,91,93,100,110)^. In contrast, 10 (32 %) found that the tools required improvement^(37,42,49,51,52,69,80,84,102,109)^, and only 3 (10 %) concluded that the tool needed to be adequate for assessing food intake in the study population^(56,86,94)^ (Table 2).

**Table 2.** Characteristics of validation studies for technologies to assess children's food intake

Brand name of technology (tool used)	Reference method	Number of days collected with the technology	Number of participants	Statistical analysis used	Participants´ characteristics (age group, sex and study site)	Principal results and conclusion	References
*Web-based Food Record* (WebFR)	Direct dietary observation	4	117	Omission rate; correspondence rate; intrusion rates	8–9 years; both sexes; Norway	Results: mean correspondence rate: 73 %, mean omission rate: 27 %, mean intrusion rate: 19 %. Conclusion: Some children had difficulty recording. Children and their parents/guardians with difficulties with the language should receive support and additional information on how to use the WebFR.	Medin *et al.*^(49)^
	Plasma carotenoid concentration	4	261	Spearman correlation coefficient; Cross-classification	8–9 years (*n* 121) and 12–14 (*n* 140) years; both sexes; Norway	Results: Spearman correlation coefficient: 0⋅30 and 0⋅44; correct classification: 71⋅6–76⋅6 %. Conclusion: WebFR was able to classify participants according to reported intake of foods high in α-carotene, β-carotene, β-cryptoxanthin and lycopene.	Medin *et al.*^(50)^
	Total energy expenditure measured by accelerometer and predictive equation	4	253	Pearson correlation coefficient; Bland–Altman analysis	8–9 years (*n* 123) and 12–14 (*n* 130) years; both sexes; Norway	Results: 36–37 % of sample underreported; 2–4 % overreported energy intake, respectively. Pearson correlation coefficient = 0⋅16. Bland–Altman analysis showed that the difference between energy intake and total energy expenditure (TEE) deviates widely from 0 and that individual underreport more than they overreport. Conclusion: Energy intake analysed by WebFR was significantly underestimated in comparison to TEE. WebFR should be used with caution in young people.	Medin *et al.*^(51)^
Portuguese self-administered computerised 24-hour dietary (PAC-24)	Direct dietary observation	1	41	Bland–Altman analysis	7–10 years; both sexes; Portugal	Results: mean correspondence rate: 67 %; mean omission rate: 21⋅5 %; mean intrusion rate: 11⋅5 %; 32 % of real intake was underestimated by PAC24. Conclusion: The tool should be validated for use in each specific population to analyse differences in accuracy between socioeconomic and racial/ethnic groups in children.	Carvalho *et al.*^(52)^
*Food Intake Recording Software System* (FIRSSt)	Direct dietary observation + 24HR	1	138	Pearson correlation coefficient and Paired *t* tests	9–11 years; both sexes; USA	Results: observation (comparison method): mean correspondence rate: 46 %; mean omission rate: 30 %; mean intrusion rate: 24 %. 24HR (comparison method); mean correspondence rate: 59 %; mean omission rate: 24 %; mean intrusion rate: 17 %. Conclusion: The tool is less accurate than 24HR for reporting a meal, but performed better for assessments throughout the day.	Baranowski *et al.*^(77)^
Web-based Dietary Assessment Software for Children (WebDASC)	Modified observation technique (photos of foods + weighing) and biomarker	7	81	Spearman correlation coefficient, intraclass correlation coefficient, kappa	8–11 years; both sexes; Denmark	Results: The tool reached 82 % agreement for school food intake, 14 % intrusions, 3 % omissions and 1 % failures for total food and beverages. Kappa agreement = 0⋅40. Conclusion: The precision for intake of fruits, juices and vegetables in WebDASC was good compared to observed intake in school meals using a digital photographic method.	Biltoft-Jensen *et al.*^(22)^
	Total energy expenditure measured by accelerometer	7	81	Pearson correlation coefficient; and Paired *t* tests; kappa; Bland–Altman analysis	8–11 years; both sexes; Denmark	Results: Proportion of participants in the same or adjoining quartile for energy was 73 %. Pearson correlation coefficient: 0⋅31. Kappa agreement: 0⋅128. Bland–Altman graph showed positive differences for energy intake but high negative differences for lower values. Conclusion: The tool is acceptable and feasible for use in collecting dietary data in school-age children.	Biltoft-Jensen *et al.*^(21)^
	Direct dietary observation with weighing; blood EPA and DHA levels	7	834	Spearman correlation coefficient, Paired *t* tests; kappa	8–11 years; both sexes; Denmark	Results: Comparing fish intake reported with EPA + DHA, correlation coefficients (*r* = 0⋅30–0⋅39) and kappa agreement (κ = 0⋅07–0⋅015). Conclusion: Accuracy was higher when all foods were assessed, compared to report of specific foods and was higher during the control period compared with the intervention period.	Biltoft-Jensen *et al.*^(23)^
	Reported whole grains *v*. plasma alkyl-resorcinol	7	750	Spearman correlation coefficient	8–11 years; both sexes; Denmark	Results: Spearman correlation coefficient: *r* = 0⋅37–0⋅40. Conclusion: Relative validity of children's self-reported intake was similar to validity of consumption reported by parents and guardians.	Biltoft-Jensen *et al.*^(24)^
*RealityMalta*	24HR	1	50	Spearman and Pearson's correlation coefficient	9–11 years; both sexes; Malta	Results: Spearman correlation coefficient: *r* = 0⋅40–0⋅63; Pearson correlation coefficient: *r* = 0⋅49–0⋅64. Conclusion: The tool can be used with reasonable confidence to assess intake of total sugars and non-milk extrinsic sugars in Maltese children.	Copperstone *et al.*^(33)^
Evernote app-based food diary	FFQ	4	16	Spearman correlation coefficient	9–12 years; both sexes; Dunedin – New Zealand	Results: Spearman correlation coefficient: *r* = −0⋅03–0⋅83. Conclusion: The tool proved promising in this study but needs formal validation in a future study.	Davison *et al.*^(38)^
*Consumo Alimentar e Atividade Física de Escolares* (Web-CAAFE)	Direct dietary observation	1	602	Poisson regression	7–11 years; both sexes; Florianópolis – Brazil	Results: High percentage of disagreement in relation to schools and types of meals. Overall agreement (43 %), intrusions (29 %) and omissions (28 %). Conclusion: Further studies are necessary to improve the validity of CAAFE.	Davies *et al.*^(37)^
	Direct dietary observation	1	390	Multinominal logistic regression	7–15 years; both sexes; Feira de Santana – Brazil	Results: mean agreement rate = 81⋅4 %; mean omission rate = 16⋅2 %; mean intrusion rate = 7⋅1 %. Conclusion: The tool was considered valid and reliable for second to fifth graders in public schools.	de Jesus *et al.*^(45)^
	Direct dietary observation	1	629	Poisson regression	7–11 years; both sexes; Santa Catarina – Brazil	Results: Moderate magnitude bias for most of the food groups. Frequency of intake did not appear to be related to this bias. Conclusion: The tool appears to be a simple, low-cost questionnaire, adaptable for assessment of school children's dietary adherence.	Kupek *et al.*^(44)^
The VioScreen FFQ	3-day estimated FR	1	55	Pearson correlation coefficient	6–14 years; both sexes; New York – USA	Results: Pearson correlation coefficients: *r* = 0⋅11–0⋅69. Conclusion: Need for modifications to adjust the questionnaire to children. Immediate next step is to perform the changes and test the FFQ again to analyse their efficacy.	Deierlein *et al.*^(84)^
*Tool for Energy Balance in Children* (TECH)	Doubly labelled water; use of KidMeal-Q (food and beverage assessment)	14	30	Paired Wilcoxon test; Spearman correlation coefficient; Bland–Altman analysis	3 years; both sexes; Sweden	Results: Spearman correlation coefficient: *r* = 0⋅42–0⋅46. Bland–Altman analysis showed overestimation for high energy intake and underestimation for low energy intake. Conclusion: One day of recording with TECH was not capable of accurately estimating energy intake or certain foods in children 3 years old.	Henriksson *et al.*^(56)^
	Double-labelled water; 24HR	4	39	Bland–Altman analysis; Spearman correlation coefficient	5–6 years; both sexes; Östergötland – Sweden	Results: Spearman correlation coefficient: *r* = 0⋅33–0⋅89. No significant differences were found in mean intake of foods using TECH and 24HR. Conclusion: The tool precisely estimated mean intake of energy and selected foods and appears to be a useful tool for dietary studies in schoolchildren.	Delisle Nyström *et al.*^(55)^
Automated Self-Administered 24-Hour Dietary Recall for Children (ASA24-Kids-2012)	Direct dietary observation	1	69	Repeated measures ANOVA; Spearman correlation coefficient	9–11 years; both sexes; Texas and Arizona – USA	Results: Texas: correspondence = 37 %; Intrusion = 27 %; omission = 35 %; Arizona: correspondence = 53 %; intrusion = 12 %; omission = 36 %; Spearman correlation coefficient. *r* = 0⋅18 (Texas); *r* = 0⋅09 (Arizona). Conclusion: The tool was less accurate than 24HR administered by interviewer when compared to observed intake, but both showed weak performance.	Diep *et al.*^(86)^
*Previous Day Food Questionnaire* (PDFQ)	Direct dietary observation	1	312	Multinominal multivariate logistic regression analysis	7–12 years; both sexes; Florianópolis – Brazil	Results: omission rate = 22⋅8 % and intrusion rate = 29⋅5 %. Conclusion: The tool showed satisfactory validity in second to fifth graders in public schools.	Engel *et al.*^(100)^
Self-Administered Children and Infants Nutrition Assessment (SACINA)	Double-labelled water	3	36	Subgroup analysis and Bland–Altman analysis	4–10 years; both sexes; Spain and Belgium	Results: near-perfect agreement was observed in lean and normal-weight children. Underestimated report = 8 %; adequate report = 78 %; overestimated report = 14 %. Conclusion: Two applications of the tool were valid for assessing energy intake in the target population group, but not at the individual level.	Börnhorst *et al.*^(102)^
Web-based automated self-administered 24-hour dietary recall (ASA24)	24HR	1	120	Correspondence, intrusion and omission rates. Multiple adjusted correlations (ANOVA and MANOVA)	8–13 years; both sexes; USA	Results: correspondence = 47⋅8 %. Omission rate = 18⋅9 %; intrusion = 12⋅5 %. Children 8 and 9 years old had substantial difficulties and often needed help. Conclusion: The authors suggest a simpler version of this tool for younger children.	Baranowski *et al.*^(80)^
Automated Self-Administered 24-Hour Dietary Assessment Tool (ASA24-Canada)	Direct dietary observation and weighing	1	40	Paired *t* tests	2–5 years; both sexes; Canada	Results: exact or proximate correspondence = 79⋅2 % (82⋅3 % lunch, 81⋅2 % snack and 77⋅4 % dinner). Conclusion: Parents are capable of report what their preschool children eat and drink with relative validity. However, the accuracy was low for estimates of meal sizes.	Wallace *et al.*^(94)^
*European Prospective Investigation into Cancer and Nutrition Software* (EPIC-Soft)	7-day FR	2	74	Wilcoxcin test; Spearman correlation coefficients	7 and 8 years (the 12–13-year age group, outside the current study's scope, was also analysed); both sexes; Denmark	Results: Spearman correlation coefficients: *r* = 0⋅27–0⋅51. Conclusion: The tool produces relatively good values for macronutrients and foods, but some differences in macronutrient intake suggest the need to adapt the tool to age and to the children's parents.	Trolle *et al.*^(42)^
No brand name (Sweden)	Paper FR and Double-labelled water	5	22	Paired *t* tests and Bland–Altman analysis	12 years; both sexes; Sweden	Results: mean energy intake from meals did not differ between the instruments. Image-assisted FR overestimated energy intake by 10 %. Conclusion: The images’ quality and parents’ prior experience with FR can bias the data. Energy expenditure was overestimated (by both this technology and the traditional method), and the lack of validation of a commercial version makes the method more expensive. Still, the technology proved more exact than common FR and is thus a more trustworthy alternative.	Johansson *et al.*^(71)^
No brand name (United Kingdom)	Direct dietary observation	5	198	Not reported	7–11 years; boys; United Kingdom	Results: accuracy = 95⋅9 %. Significant discrepancy was observed between what the researcher reported and what the smart card recorded. Conclusion: The tool showed power for monitoring the choice of foods/nutrients for a limited time in school dining halls.	Lambert *et al.*^(62)^
No brand name (USA)	24HR	7	26	Spearman correlation coefficient	9–12 years; both sexes; California – USA	Results: Spearman correlation coefficient: *r* = 0⋅62 (grains) and *r* = 0⋅78 (fruits). Conclusion: The results showed potential for use of the FR based on digital images to assess one day intake of food groups.	Matthiessen *et al.*^(91)^
No brand name (USA)	Weighting leftover food and Direct dietary observation	4	Not informed	Pearson correlation coefficient; Paired *t* tests; Bland–Altman analysis	3rd to 5th graders (age not informed); both sexes; USA	Results: Pearson correlation coefficient: *r* = 0⋅96 (digital image *v*. weighing); *r* = 0⋅98 (digital image *v*. direct observation); no statistically significant difference was observed in mean consumption of fruits and vegetables in digital images *v*. reference methods. Conclusion: The tool was valid for assessing mean consumption, but less accurate for estimating consumption of food served on trays.	Taylor *et al.*^(93)^
Synchronised Nutrition and Activity Program™ (SNAP™)	24HR	1	121	Bland–Altman analysis; differences in mean frequency of intake	7–15; both sexes; England	Results: The tool underestimated the mean counts of dietary components. Conclusion: The tool is a rapid, precise and low-cost method at the population level.	Moore *et al.*^(67)^
No brand name (United Kingdom)	FR	2	78	Paired *t* tests; kappa agreement	7–15; both sexes; England	Results: kappa agreement: kappa = 0⋅00–0⋅29. Conclusion: More studies are necessary to determine whether the questionnaire can be modified to improve the precision in the way children report the items that they ate outside of school.	Moore *et al.*^(69)^
Digital photography (no brand name)	24HR	4	40	Paired *t* tests	7–8; both sexes; Turkey	Results: There was a statistically significant difference in the mean energy intake according to the technology and the reference method in both sexes. Conclusion: The digital method did not show any advantage in terms of its accuracy, but it did show greater ease of adherence at lower ages.	Erkilic *et al.*^(109)^
	24HR	3	21	Spearman correlation coefficient; Bland–Altman analysis	5–7; both sexes; Sweden	Results: Spearman correlation coefficients: *r* = 0⋅60–0⋅84 Bland–Altman: underestimation in all the food groups analysed. Conclusion: The tested method was considered acceptable and accessible.	Norman *et al.*^(110)^

FR, Food Records; 24HR, 24-hour dietary recall; App, Application; USA, United States of America; FFQ, Food Frequency Questionnaire; ANOVA, Analysis of Variance; MANOVA, Multivariate Analysis of Variance.

## Discussion

As far as we know, the present study was the first scoping review on technologies developed to assess children's food intake. Most of the technologies analysed had the following characteristics: web-based software packages; developed for children over 6 years of age; assessed food intake with 24HR and collected data at home. Most were self-administered; used text-based data entry based on a list of predefined names/categories of foods; published in English; allowed assessing the amount consumed; estimated food portions with photographs of foods; assessed energy and nutrient intake; did not report assessment of supplement intake and did not report whether they included a food composition database. These results corroborate the review by Eldridge *et al.*, which found that of fourteen technological tools used only to assess children's food intake, 50 % were based on 24HR; 50 % were web-based and 43 % were developed in Europe^(2)^. Meanwhile, a systematic review by Kouvari *et al.* found that 91 % of the eleven technologies used for the same purpose were web-based^(7)^.

The Danish software Web-DASC was the most widely analysed technology in the publications, totalling nine studies referring to the tool^(17–25)^, with four of these studies assessing its validation^(21–24)^. Web-DASC is a web-based software featuring a list of foods and beverages with around 1300 items self-administered by children with or without help from parents or guardians. The age group cited by all the studies involving this technology was 8–11 years^(17–25)^. The second most frequently cited technology in the studies was NDSR, from the USA, mentioned in seven studies^(26–32)^, and no study that assessed its validation was found. This software has an offline feature, and unlike Web-DASC, the food intake report is obtained with an interview. The list of foods and beverages in this technology is longer, with 1800 food items, and the age group mentioned by the various studies ranged from 0 months to 11 years of age^(26–32)^. Other technologies frequently mentioned in the studies were Web-CAAFE and *EPIC-Soft*. The former, developed in Brazil, was cited in five articles^(44–48)^, including three validation studies^(37,44,45)^. This software is web-based, self-administered by children 7–15 years of age, and features a list of 300 groups of foods and beverages^(44–48)^. Meanwhile, the EPIC-Soft version for children was developed jointly between Denmark and the Basque region. It is software with offline functionality, in which data are collected via an interview with the child and a parent or guardian^(39–43)^. As with Web-CAAFE, five studies cited EPIC-Soft^(39–43)^, and one focused on its validity^(42)^. No information was found on the amounts of foods and beverages in this software, and three age groups were analysed: 4–5, 7–8 and 12–13 years^(39–43)^.

Although our review identified technological resources that are currently no longer applied to the methodology for assessing children's food intake, such as digital cameras, which are now integrated into smartphones, providing much simpler digital food item image snapshots^(2)^, other tools caused a stir because of the innovations employed as assessment methods. Two studies reported technologies with smartcard systems. Lambert *et al.*^(62–64)^ performed three simultaneous studies to develop and validate the use of cards that functioned with the same principle as a debit card, in which the foods purchased by the children were recorded in a databank. Luszczki *et al.*^(66)^ used cards with barcodes to identify children that used the card to purchase fruits and vegetables at school. None of the studies that approached these technologies named the tools. No validation study was retrieved on the technology analysed by Luszczki *et al.*^(66)^. Another technological innovation presented by Beltran *et al.*^(82,83)^ was on eButton. This portable device was attached to children's clothing at chest level, using a multi-sensor camera to capture data on the foods and beverages consumed. The foods were identified by nutritionists using images obtained by the device, and a three-dimensional digital mesh procedure quantified the portions. No validation study was found for this technology either.

An essential aspect in the technologies’ development was that most failed to inform whether the technological resources presented databases on the foods’ nutritional composition integrated into the tool. However, most allowed estimating energy and nutrient intake, which suggests that the estimates are performed separately by the technology via data analysis using information from foods’ nutritional composition tables. The children's diverse diets could hinder the automatic integration of data on the foods’ nutritional composition into these technological tools. Still, automated information would facilitate data analysis and the elaboration of real-time feedback on energy and nutrient intake from children or guardians^(111)^. Of the technologies assessed in the present study, TECH^(55)^, Web-DASC^(17)^, NDSR^(26)^ and Button^(82)^ have food composition databases integrated into the tool.

Another apparently non-trivial aspect revealed by our study is the analysis of children's food intake in their respective settings, namely at school and home. We should ideally analyse both settings to have a reliable measure of children's dietary intake, contrary to the current review, in which fewer than 10 % of the studies analysed both settings. Studies in only one of the food settings pose a limitation for a more comprehensive and detailed understanding of the sample (so that it does not actually represent 24-hour intake)^(37)^. The setting may also be limited in terms of the variety of foods offered, while parents’ control of their children's diet influences the children's food choices^(44)^ in different food contexts.

The search identified only one technology developed for a Latin American population group, called Web-CAAFE, a software for qualitative assessment of Brazilian children's dietary intake^(44)^. Furthermore, only one technology was found for children's food intake in Africa: Zambia Tablet-based 24 h Recall Tool. These findings may be explained by the shortage of funding for the development of this kind of tool in low- and middle-income countries^(99)^.

Since the use of a technology developed for a different population is limited by language and the cultural eating specificities, the current review highlights the need for funding to develop tools to assist data collection on children's dietary intake, thus fomenting studies in food, nutrition and nutritional epidemiology, which also emphasises the need to investigate the usability of these technological tools in specific population groups, such as economically underprivileged children and their parents or those with low schooling^(99)^.

The current scoping review has some limitations regarding accessing specific studies that still need to be retrieved despite attempts to contact the authors. Another issue was the data extraction stage. We observed a need for more information on some characteristics of the respective technological tools obtained in some situations through cross-references. On the other hand, the scoping review was designed and conducted according to the *Joanna Briggs Institute Reviewer Manual*^(16)^ to minimise potential biases. We also opted for a high-sensitivity search strategy, allowing an expanded search for relevant articles.

## Conclusions

We believe that the current review provided relevant and sufficient information on the existing technologies for assessing children's food intake, allowing us to summarise helpful information for studies that are intended to use the existing tools and those intended to develop or validate tools with several innovations and targeted to places with a shortage of such technologies.

## Supplementary material

For supplementary material accompanying this paper visit https://doi.org/10.1017/jns.2023.27. click here to view supplementary material
