# Supplementary material for: Technological tools for assessing children's food intake: a scoping review
Source: J Nutr Sci. 2023 Apr 11;12:e43. doi: 10.1017/jns.2023.27 (PMC10131056; doi:10.1017/jns.2023.27)
Supplement: Supplementary file 1 [file jnssup.zip › S2048679023000277sup002.docx]

Supplementary Table 3. Database search strategy.

| Pubmed (5.673) | #1: child[mesh] OR child*[tiab] OR Child, Preschool[Mesh] or infant[mesh] OR infant*[tiab] OR pre-school*[tiab] OR preschool*[tiab] OR young*[tiab] OR adolescent[Mesh] or adolescent*[Tiab]  #2: software[Mesh] OR Software*[Tiab] OR Software Design[Mesh] OR Software Validation[Mesh] OR Technology[Mesh] OR Technologies*[Tiab] OR Computer[Mesh] OR Computer*[Tiab] OR Cell Phone[Mesh] OR Cell Phone[Tiab] OR Mobile*[Tiab] OR Computers, Handheld[Mesh] OR Mobile application[Mesh] OR Smartphone[Mesh] OR Smartphone*[tiab] OR Personal Digital Assistant[tiab] OR Pocket PC[tiab] OR Palm Pilot*[tiab] OR Microcomputers[Mesh] OR microcomputer*[tiab] OR Microprocessor*[tiab] OR Decision Making, Computer-Assisted[Mesh] OR “electronic questionnaire”[tiab] OR “web questionnaire”[tiab]OR “web-based”[tiab] OR “internet-based”[tiab]  #3: Diet[Mesh] or Diet[tiab] or Diets[tiab] or Dietar[tiab] or Food Consumption[mesh] or Food Consumption[tiab] or Dietary pattern[tiab] or Habit Diet[tiab] or Eating[Mesh] OR Eaten[tiab] OR Eating[Tiab] or Food intake[Tiab] OR  Feeding Patterns[tiab] OR Food habits[tiab] OR nutrition assessment[Mesh] OR nutrition assessment[Tiab] OR nutrition measurement[tiab]  Search: #1 AND #2 AND #3 |
| --- | --- |
| Scopus (1.629) | #1: ( TITLE-ABS-KEY ( ( *child**  OR  *"child preschool"*  OR  *infant**  OR  *pre-school**  OR  *preschool**  OR  *young**  OR  *adolescent** ) ) )#2 ( ( TITLE-ABS-KEY ( ( *software**  OR  *technolog**  OR  *computer**  OR  *"Cell Phone"*  OR  *mobile**  OR  *smartphone**  OR  *"Personal Digital Assistant"*  OR  *"Pocket PC"*  OR  *palm*  AND  *pilot**  OR  *microcomputer**  OR  *microprocessor** ) )  OR  TITLE-ABS-KEY ( ( *"Decision Making Computer-Assisted"*  OR  *"electronic questionnaire"*  OR  *"web questionnaire"*  OR  *"web-based"*  OR  *"internet-based"* ) ) ) )#3 ( TITLE-ABS-KEY ( ( *diet*  OR  *diets*  OR  *dietary*  OR  *"Food Consumption"*  OR  *"Dietary pattern"*  OR  *eating*  OR  *eaten*  OR  *"Food intake"*  OR  *"Feeding Pattern"*  OR  *"Food habits"*  OR  *"nutrition assessment"*  OR  *"nutrition measurement"* ) ) ) Search: #1 AND #2 AND #3 |
| Web of science (5.073) | **#1: TÓPICO:**(child* OR “child, preschool” OR infant* OR pre-school* OR preschool* OR young* OR adolescent*)  **#2: TÓPICO:** (Software* OR Technolog* OR Computer* OR “Cell Phone” OR Mobile* OR Smartphone* OR “Personal Digital Assistant” OR “Pocket PC” OR Palm Pilot* OR Microcomputer* OR Microprocessor* OR “Decision Making, Computer-Assisted” OR “electronic questionnaire” OR “web questionnaire” OR “web-based” OR “internet-based”)  **#3: TÓPICO:** (Diet or Diets or Dietary OR “Food Consumption” OR “Dietary pattern” OR Eating OR Eaten OR “Food intake” OR “Feeding Pattern” OR “Food habits” OR “nutrition assessment” OR “nutrition measurement”) |
| Cochrane (2.295) | #1 MeSH descriptor: [Child] explode all trees 58731  #2 MeSH descriptor: [Child, Preschool] explode all trees 30532  #3 MeSH descriptor: [Infant] explode all trees 33554  #4 MeSH descriptor: [Adolescent] explode all trees 107314  #5 (child* OR “child, preschool” OR infant* OR pre-school* OR preschool* OR young* OR adolescent*):ti,ab,kw 360338  #6 #1 or #2 or #3 or #4 or #5 360338  #7 MeSH descriptor: [Software] explode all trees 3834  #8 MeSH descriptor: [Software Design] explode all trees 82  #9 MeSH descriptor: [Software Validation] explode all trees 27  #10 MeSH descriptor: [Technology] explode all trees 6075  #11 MeSH descriptor: [Computers] explode all trees 1927  #12 MeSH descriptor: [Computers, Handheld] explode all trees 852  #13 MeSH descriptor: [Smartphone] explode all trees 476  #14 MeSH descriptor: [Microcomputers] explode all trees 1097  #15 MeSH descriptor: [Decision Making, Computer-Assisted] explode all trees 4336  #16 ((Software* OR Technolog* OR Computer* OR “Cell Phone” OR Mobile* OR Smartphone* OR “Personal Digital Assistant” OR “Pocket PC” OR “Palm Pilot” OR Microcomputer* OR Microprocessor* OR “Decision Making, Computer-Assisted” OR “electronic questionnaire” OR “web questionnaire” OR “web-based” OR “internet-based”)):ti,ab,kw 102082  #17 #7 OR #8 OR #9 OR #10 OR #11 OR #12 OR #13 OR #14 OR #15 OR #16 107415  #18 MeSH descriptor: [Diet] explode all trees 19497  #19 MeSH descriptor: [Eating] explode all trees 3784  #20 MeSH descriptor: [Nutrition Assessment] explode all trees 714  #21 ((Diet or Diets or Dietary OR “Food Consumption” OR “Dietary pattern” OR Eating OR Eaten OR “Food intake” OR “Feeding Patterns” OR “Food habits” OR “nutrition assessment” OR “nutrition measurement”)):ti,ab,kw 107784  #22 #18 OR #19 OR #20 OR #21 110847  #23 #6 AND #17 AND #22 2295 |
| LILACS via VHL (441) | #1: (mh:(adolescent)) OR (mh:(infant)) OR (mh:(Child, Preschool)) OR (mh:(child)) OR (tw:(child*)) OR (tw:(infant*)) OR (tw:(pre-school*)) OR (tw:(preschool*)) OR (tw:(adolescent*)) OR (tw:( young*))  #2: (mh:(software)) OR (mh:("Software Design")) OR (mh:("Software Validation")) OR (mh:(Technology)) OR (mh:(Computer)) OR (mh:("Computers, Handheld")) OR (mh:("Mobile application")) OR (mh:(Smartphone)) OR (tw:(Software*)) OR (tw:(Technolog*)) OR (tw:(Computer*)) OR (tw:("Cell Phone")) OR (tw:(Mobile)) OR (tw:(Smartphone*)) OR (tw:("Personal Digital Assistant")) OR (tw:("Pocket PC")) OR (tw:(“Palm Pilot”)) OR (mh:(Microcomputers)) OR (mh:(Decision Making, Computer-Assisted)) OR (tw:(Microprocessor*)) OR (tw:(“electronic questionnaire”)) OR (tw:(“web questionnaire”)) OR (tw:(“web-based”)) OR (tw:(“internet-based”))  #3:(mh:(Diet)) OR (mh:(Food Consumption)) OR (mh:(Eating)) OR (mh:(nutrition assessment)) OR (tw:(Diet)) OR (tw:(Diets)) OR (tw:(Dietary)) OR (tw:("Food Consumption")) OR (tw:("Dietary pattern")) OR (tw:(Habit Diet*)) OR (tw:(Eaten)) OR (tw:(Eating)) OR (tw:("Food intake")) OR (tw:(“Feeding Patterns”)) OR (tw:(“Food habits”)) OR (tw:("nutrition assessment")) OR (tw:("nutrition measurement"))  Search: #1 AND #2 AND 3 |
| OpenGrey (8) | (child OR adolescent) AND (Smartphone OR software OR Technology OR computer OR smartphone) AND (diet OR "Food Consumption" or “Food intake” OR Food habit* OR “nutrition assessment”) |
