# Supplementary material for: Technological tools for assessing children's food intake: a scoping review
Source: J Nutr Sci. 2023 Apr 11;12:e43. doi: 10.1017/jns.2023.27 (PMC10131056; doi:10.1017/jns.2023.27)
Supplement: Supplementary file 1 [file jnssup.zip › S2048679023000277sup003.docx]

**Supplementary Table 2. Eligibility criteria for study selection.**

| ***Inclusion Criteria*** | ***Exclusion Criteria*** |
| --- | --- |
| 1. Study that uses technological tool for children’s (0-9y) dietary intake assessment. | 3. Study that investigate children’s (0- 9y) dietary intake assessment using traditional method (in paper) or use technological tool exclusively to analyze data. |
| 2. Development,validation or calibration studies of technological tool for children’s (0-9y) dietary intake assessment. | 4. Study that use the technological tool to nutritional  education or to promote healthy food habits or weight management (mainly clinical trials) or food behaviors or food preferences. |
|  | 5. Study that exclusively evaluated breastfeeding. |
|  | 6. Systematic Review. |
|  | 7. Study protocol or editorials. |
|  | 8. Conference abstract or poster abstract only. |
|  | 9. Not enough information to describe the technology/insufficient information for inclusion. |
|  | 10. Articles written in other language than English, Portuguese, and Spanish. |
