# Supplementary material for: Technological tools for assessing children's food intake: a scoping review
Source: J Nutr Sci. 2023 Apr 11;12:e43. doi: 10.1017/jns.2023.27 (PMC10131056; doi:10.1017/jns.2023.27)
Supplement: Supplementary file 1 [file jnssup.zip › S2048679023000277sup004.docx]

| Supplementary Table 4. Summary of full text records non-selected and principal reason(s) for exclusion (*n*=186). | | |
| --- | --- | --- |
| **First Author, year** | **Reference** | **Numbering of the inclusion or exclusion criteria used to exclude the article according to Supplementary Table 1.** |
| Abranches AD et al., 2018 | J Pediatr. (Rio J). 2018; 94(6):652-7. | 3 |
| Adamson AJ and Baranowski T, 2014 | J Hum Nutr Diet. 2014; 27 Suppl 1:1-4. | 7 |
| Agbozo F et al., 2018 | Nutr Health. 2018; 24(4):241-49. | 3 |
| Ahmadi A et al., 2014 | J Pak Med Assoc. 2014; 64(3):316-21. | 3 |
| Ahmed B et al., 2017 | Ann Nutr Metab. 2017; 71 (suppl 2):467. | 3, 8 |
| Ahmed A and Ouzzani M, 2013 | Matern Child Health J. 2013; 17:809–15. | 5 |
| Akmal Muhamat N et al., 2021 | PLoS One. 2021;16(9):e0257035 | 4 |
| Ambroszkiewics J et al., 2011 | Medycyna wieku rozwojowego. 2011;15(3):326-34. | 10 |
| Andersen LF et al, 2011 | Eur J Clin Nutr. 2011;65(1): S58-64. | 9 |
| Andersen R et al, 2013 | Ann Nutr Metab. 2013;63(suppl 1):197 | 8 |
| Arechavala T et al, 2017 | Food Nutr Res. 2017;61(1):1391665 | 3 |
| Assis MAA et al, 2009 | Cad. Saúde Pública. 2009 ;25(8) :1816-26 | 9 |
| Bajaj D et al., 2017 | 8th International Conference on Computing, Communication and Networking Technologies (ICCCNT).  IEEE, 2017. p. 1-7. | 4 and the study does not reach the inclusion criteria 1 |
| Ball S et al., 2008 | J Am Diet Assoc. 2008; 108(4):718-21. | 3 |
| Balter KM, 2009 | Ann Nurt Metab. 2009;55:468 | 8 |
| Balthazar E and Oliveira M, 2011 | BMC Res Notes. 2011; 4(1): 1-7 | 3 |
| Barja-Fernández S et al., 2020 | Nutr Hosp. 2020;37(1):93-100 | 3 |
| Bel S et al., 2016 | Arch Public Health. 2016; 74(1):1-11 | 7 |
| Bell BM et al., 2018 | Games for Health Journal. 2018; 7(2): 127-35 | 4 |
| Bel-Serrat S et al., 2014 | Public Health Nutr. 2014;17(2):266-76. | 3 |
| Bernard L et al., 1995 | J Am Diet Assoc. 1995;95(7):800-02 | 3 |
| Bernardi JL et al., 2003 | Sao Paulo Med J. 2003;121(1):5-8 | 3 |
| Biro G et al, 2002 | Eur J Clin Nutr. 2002;56(2): S25-32. | 9 and the study does not reach the inclusion criteria 1 |
| Bischoff AR, 2018 | Br J Nutr. 2018;119(11):1295-1302 | 9 |
| Blanchet R et al., 2014 | Public Health Nutr. 2014;17(12):2844-52 | 3 |
| Blaney S et al., 2019 | Nutrients. 2019;11(3):685. | 3 |
| Boggio V & Klepping J, 1981 | Arch Fr Pediatr. 1981;38(9):679-86. | 10 |
| Boushey CJ et al., 2009 | Eur J Clin Nutr. 2009;63(Suppl 1):S50–S57 | The study does not reach the inclusion criteria 1 |
| Briassoulis G and Meyer R, 2015 | Pediatr Crit Care Med. 2015;16(6):596-98. | 7 |
| Brown AC and Brenton B, 1994 | J Am Diet Assoc. 1994;94(5):517-22. | 3 and the study does not reach the inclusion criteria 1 |
| Brunner TA et al., 2018 | Eur J Nutr. 2018;57(7):2489–99. | 3 |
| Buonomo E et al., 2012 | Int. J. Environ. Res. Public Health. 2012;9(2):421-34. | 3 |
| Brussaard JH et al, 2002 | Eur J Clin Nutr. 2002;56(2): S89-94 | 9 |
| Cadario F et al., 2012 | J Endocrinol Invest. 2012;35(2):160-68. | 3 |
| Caetano MC et al., 2010 | J Pediatr (Rio J). 2010;86(3):196-201. | 3 |
| Caetano MC et al., 2009 | J Pediatr (Rio J). 2009;85(6):509-15. | 3 |
| Calvo-Lerma J et al, 2019 | J Acad Nutr Diet. 2019;119(8):1305-19 | 3 |
| Carruth BR and Skinner JD, 2000 | J Am Coll Nutr. 2000;19(6):771-80. | 3 |
| Caselli TB et al., 2017 | Arq Gastroenterol. 2017;54(4):292-96. | The study does not reach the inclusion criteria 1 |
| Caetano MC et al., 2012 | Rev Soc Boliv Pediatr. 2012;51(2):141-48. | 3 |
| Champagne CM et al., 2004 | J Am Diet Assoc. 2004;104(2):199-207. | 3 |
| Clifton PM et al, 2011 | Nutr Metab (Lond). 2011;8(1):1-11 | 9 |
| Chlebna-Sokol D & Blaszczyk A, 2003 | Medycyna wieku rozwojowego. 2003;7(2):173-80. | 10 |
| Chlebna-Sokol D et al., 2003 | Przeglad lekarski. 2003;60(Suppl 6):60-64. | 10 |
| Cullen KW, Baranowski T and Baranowaki J, 1998 | J Nutr Educ. 1998;30(6):405-09. | The study does not reach the inclusion criteria 1 |
| Cullen KW et al., 2015 | J Acad Nutr Diet. 2015;115(5):743-50. | 3 |
| Cullen KW,Thompson D and Chen T, 2017 | Health Educ Behav. 2017;44(1):32-40. | 4 |
| Cunha LR, da Costa THM and Caldas ED, 2013 | Biol Trace Elem Res. 2013;151(1):30–37. | 3 and the study does not reach the inclusion criteria 1 |
| da Silva AC, Tavares MS and Penido MGMG, 2016 | World J Nephrol. 2016;5(6):507-16. | The study does not reach the inclusion criteria 1 |
| (da) Silva JVL et al., 2010 | Rev Bras Epidemiol. 2010;13(1)83-93. | 3 |
| Danekova N et al., 2008 | Arch Pediatr. 2008;15(8):1263-69 | 10 |
| Davies VF et al., 2015 | J Hum Nutr Diet. 2015;28(Suppl 1):65-72. | The study does not reach the inclusion criteria 1 |
| (de) Jager I et al., 2019 | Nutr J. 2019;18(1):1-16. | 3 |
| (de) Jesus GM et al., 2016 | Rev Bras Med Esporte [online]. 2016;22(4):261-66. | The study does not reach the inclusion criteria 1 |
| Dennison BA et al, 1999 | J Am Coll Nutr. 1999;18(4):346-52 | 3 |
| Densupsoontorn N et al.,2019 | Asia Pac J Clin Nutr. 2019;28(1):116-21. | 3 |
| Don T, Friedlander S and Wong W, 2010 | J Ren Nutr. 2010;20(1):23-28. | 3 |
| Fernandez-Luque et al., 2017 | BMC Med Inform Decis Mak. 2017;17:37 | 4 |
| Filigno SS et al., 2016 | In: Pediatric pulmonology. 111 river st, hoboken 07030-5774, nj usa: wiley-blackwell, 2016: 51:421-421 | 3, 8 |
| Foster E et al, 2008 | Br J Nutr. 2008;99(1):185-90 | 3 |
| Foster E et al, 2008 | Br J Nutr. 2008);99(1): 175–184 | The study does not reach the inclusion criteria 1 |
| Foster E et al, 2013 | J Hum Nutr Diet. 2013;27(1):18-25 | The study does not reach the inclusion criteria 1 |
| Forbes LE et al, 2009 | Appl Physiol Nutr Metab. 2009;34(4):648-58. | The study does not reach the inclusion criteria 1 |
| Freiberg CK et al, 2012 | Rev Assoc Bras Nutr. 2012;4(5):17-21 | 3 |
| Gamboa-Delgado EM et al., 2007 | Rev salud pública. 2007;9(1):129-39. | 3 |
| Garriguet D, 2007 | Health reports. 2007;18(2):17-32. | 3 |
| Garriguet D, 2009 | Health reports. 2009;20(3):41-52. | 3 |
| Gates M et al., 2013 | J Nutr Educ Behav. 2013;45(5):455-59. | The study does not reach the inclusion criteria 1 |
| Gomez SF et al., 2014 | BMC Pediatr. 2014;14(1):1-6. | 7 |
| Grajeta H et al., 2003 | Roczniki Panstwowego Zakladu Higieny. 2003;54(4):417-25. | 10 |
| Groele B et al., 2018 | Int J Environ Res Public Health. 2018;15(12):2833. | The study does not reach the inclusion criteria 1 |
| Groele B et al., 2019 | Sustainability. 2019;11(12): 3398. | The study does not reach the inclusion criteria 1 |
| Hamułka J, gronowska-Senger A and Witkowska K, 2000 | Rocz Panstw Zakl Hig. 2000;51(3):279-90 | 10 |
| Hanning RM et al., 2019 | Int J Environ Res Public Health. 2019;16(14):2563. | The study does not reach the inclusion criteria 1 |
| Hari A et al., 2015 | Pan Afr Med J. 2015; 20:244. | 3 |
| Harvey L, Bryant-Waugh R and Watkins B, 2015 | J Child Health Care. 2015;19(3):392-401. | 3 |
| Helle C , Hillesund ER and Øverby NC, 2018 | PLoS One. 2018;13(6):e0199455 | 9 |
| Helle C et al., 2019 | Int J Behav Nutr Phys Act. 2019;16(1):1-16 | 4 |
| Himes JH et al, 2003 | Preventive Medicine. 2003; 37(1): S55–S61 | 3 |
| Hill KM et al., 2012 | J Food Sci. 2012;77(8):H170-75. | 3 |
| Huybretchts I et al., 2010 | Appetite. 2010;54(2):340-45. | 3 |
| Huysentruyt K et al., 2016 | In: Annual Meeting of the European Society for Paediatric Gastroenterology, Hepatology and Nutrition (ESPGHAN). Conference publication: (varpagings). 2016;62:700. | 3, 8 |
| Huysentruyt K et al., 2015 | Eur J Nutr. 2015;55(4):1595-604. | 3 |
| Jaramillo SJ et al., 2006 | J Nutr Educ Behav. 2006;38(6):352-59. | 4 |
| Jati IRAP et al., 2014 | Nutrition. 2014;30(11-12):1310-17. | The study does not reach the inclusion criteria 1 |
| Jesus GM et al., 2017 | Rev Bras Epidemiol. 2017;20(4):573-85 | The study does not reach the inclusion criteria 1 |
| Johnson CC et al., 2017 | Int J Environ Res Public Health. 2017; 14(4):415. | The study does not reach the inclusion criteria 1 |
| Kang M et al., 2016 | J Acad Nutr Diet. 2016;116(8):1316-22. | 3 |
| Kehoe L et al., 2016 | J Hum Nutr Diet. 2017;30(4):405-16. | 3 |
| Kim EJ et al., 2014 | Child Care Health Dev. 2014;40(5):698-705. | 3 |
| Kroller K, Kroller A and Warschburguer P, 2013 | Zeitschrift Fur Gesundheitspsychologie. 2013;21(2):53-61. | 10 |
| Kupek E and de Assis MA, 2016 | British Journal of Nutrition. 2016; 116(5): 904-912. | 3 |
| Lacerda EMA et al., 2021 | Cad. Saúde Pública. 2021;37(8):e00301420 | 9 |
| Lagstrom H et al, 1997 | Arch Pediatr Adolesc Med. 1997;151(2):181-88. | 4 |
| Liu P et al., 2010 | Zhonghua yu fang yi xue za zhi. 2010;44(3):204-08. | 10 |
| Lanigan JA et al., 2001 | Eur J Clin Nutr. 2001;55(2):124-29. | 3 |
| Lee RM et al., 2014 | Int J Behav Nutr Phys Act. 2014;11(1):1-9. | The study does not reach the inclusion criteria 1 |
| Leung SSF et al., 2001 | J. Paediatr. Child Health. 2001; 37(3), 247–53 | 3 |
| Leung TF et al., 2017 | Hong Kong medical journal = Xianggang yi xue za zhi. 2017;23(5):470-79 | 3 |
| Lima ACS et al., 2017 | Braspen J. 2017;32(2):134-39. | 3 |
| Longo-Silva G et al., 2012 | Rev paul pediatr. 2012;30(1):35-41. | 3 |
| Longo-Silva G et al., 2014 | Rev paul pediatr. 2014;32(2):193-99. | 3 |
| Looney SM and Raynor HA, 2012 | Int J Environ Res Public Health. 2012; 9(4): 1368–78 | 3 |
| Lopes C et al, 2018 | JMIR Res Protoc. 2018 Feb 15;7(2):e42. | 7 |
| Luna RCP et al., 2011 | Rev Inst Adolfo Lutz. 2011;70(2):213-19. | 3 |
| Lupinska A and Chlebna-Sokół D, 2017 | Pediatr Med Rodz. 2017;13(4):514–26. | 3 |
| Luque V et al., 2013 | J Pediatr Gastroenterol Nutr. 2013;56(3):320-27. | The study does not reach the inclusion criteria 1 |
| Maalouf J et al., 2013 | Child Obes. 2013;9(5):437-45. | 3 |
| Mak TN et al., 2013 | PHN. 2013;16(11):1912-23. | 3 |
| Maneschy IR, 2015 | Faculdade de Medicina da Universidade de São Paulo. Programa de Pediatria. 2015 [Dissertação (mestrado)] | 3 |
| Marcinek K, Wójciak RW and Krejpcio Z, 2016 | Rocz Panstw Zakl Hig. 2016;67(2):169-77. | 3, 12 |
| Marreiro DN, Fisberg M and Cozzolino SMF, 2002 | Biol Trace Elem Res. 2002;86(2):107-22. | The study does not reach the inclusion criteria 1 |
| Marreiro DN, Fisberg M and Cozzolino SMF, 2004 | Biol Trace Elem Res. 2004;100(2):137-49. | The study does not reach the inclusion criteria 1 |
| Martin CL, Murphy SP and Novotny R, 2008 | J Am Diet Assoc. 2008;108(11):1874-80. | 3 |
| Martínez AB et al., 2010 | Nutr Hosp. 2010;25(3):394-99. | 3 and the study does not reach the inclusion criteria 1 |
| Marty L et al., 2017 | Front Nutr. 2017; 4:16. | 4 |
| McAfee AJ et al., 2012 | PHN. 2012;15(9):1670-77. | 3 |
| Mensink GBM et al., 2007 | Bundesgesundheitsblatt Gesundheitsforschung Gesundheitsschutz. 2007;50(5-6):902-08. | 10 |
| Merkiel S and Chalcarz W, 2016 | Rocz Panstw Zakl Hig. 2016;67(2):179-88. | 3 |
| Merkiel-Pawłowska S and Chalcarz W, 2017 | BMC Pediatr. 2017;17(1):1-11. | 3 |
| Meyer R et al., 2015 | Clin Transl Allergy. 2015;5(1):1-7. | 3 |
| Mitchell DC et al, 2000 | Journal of Nutrition Education, 32(2), 100-03. | 3, 4 |
| Moore L and Tapper K, 2008 | Journal of Epidemiology & Community Health, 2008; 62(10), 926-31. | 9 |
| Moraeus L et al., 2018 | Food Nutr Res. 2018; 62:1-10. | 7 and the study does not reach the inclusion criteria 1 |
| Morrissey B, Allender S and Strugnell C, 2019 | Int J Environ Res Public Health. 2019;16(10):1778. | 4 |
| Ndungu ZW and Chege PM, 2019 | Nutrition and Food Technology: Open Access. 2019;5(1):1-5. | 3 |
| Nicklas T et al, 2017 | International Journal of Behavioral Nutrition and Physical Activity, 2017: 14(1), 1-10. | 9 |
| Ning Y et al, 2018 | Biol Trace Elem Res. 2018;183(2):233-44. | 3 |
| Ning Y et al, 2018 | J Trace Elem Med Biol. 2018; 50:441-60. | 3 |
| Novotny R et al, 2013 | Food chemistry. 2013;140(3):471-77. | The study does not reach the inclusion criteria 1 |
| O’Brien N, Roe C and Reeves S, 2002 | HEJ. 2002;61(4):320-28. | 3 |
| Ocke M et al., 2015 | Eur J Nutr. 2015;54(5):721-32. | 3 |
| Oreskovic NM et al., 2016 | Contemp Clin Trials. 2016;49:149-54. | 4 |
| Pani P et al., 2014 | BMC Pediatr. 2014;14(1):1-8. | 3 |
| Perales-Garcia A et al, 2018 | PloS one. 2018; 13(12): e0208748 | 3 |
| Pereira AS et al, 2013 | Cad saúde colet. 2013;21(2):140-47. | 3 |
| Pereira AM et al, 2000 | J Ren Nutr. 2000;10(1):24-29. | 3 |
| Piple J et al, 2015 | PloS one. 2015;10(11):e0140807. | 3 |
| Powers SW et al, 2016 | In: Pediatric pulmonology Conference: 30th annual north american cystic fibrosis conference United states. Conference publication: 2016;51:418‐19. | 3, 8 |
| Randecker GA et al, 1996 | *Diabetes Care*. 1996; *19*(12): 1370-1374. | 3 |
| Raymond J et al, 2017 | Arch Public Health. 2017;75(1):1-8. | 3 |
| Robson SM et al, 2019 | International Journal of Behavioral Nutrition and Physical Activity. 2019; 16(1): 1-9. | 3 |
| Rockell JE et al, 2011 | Public health nutrition. 2011; 14(2): 203-208. | 9 |
| Rodan MF et al, 1997 | Am J Clin Nutr. 1997;65(4):s1311. | The study does not reach the inclusion criteria 1 |
| Rosell et al., 2021 | Acta Pediatr. 2021;110(9):2597-2606 | 9 |
| Ruggeri BFF, 2011 | USP [Tese], 2011:115. | 7 and the study does not reach the inclusion criteria 1 |
| Ruggeri BFF et al, 2012 | Nutrire Rev Soc Bras Aliment Nutr. 2012;37(3):309-21. | 7 and the study does not reach the inclusion criteria 1 |
| Rusińska A et al, 2011 | Pediatr Endocrinol Diabetes Metab. 2011;17(2):82-87. | 10 |
| Salles-Costa R et al, 2007 | Rev bras epidemiol. 2007;10(2):267-75. | 3 |
| Salvo D et al, 2012 | Nutr J. 2012;11(1):1-9. | 3 |
| Schiel R, Kaps A and Bieber G, 2012 | Appetite. 2012;58(2):432-37. | 4 and the study does not reach the inclusion criteria 1 |
| Serra-Majem L et al, 2004 | Public Health Nutr. 2004;7(7):931–35. | 9 |
| Shamah-Levy T et al, 2016 | J Nutr. 2016;146(5):1043-50. | 3 |
| Sharkey JR et al, 2012 | BMC Pediatr. 2012;12(1):1-12. | 3 |
| Shatenstein B et al, 2010 | J Pediatr Gastroenterol Nutr. 2010;51(5):645-52. | 3 |
| Silva BNS et al, 2017 | Rev Nutr (Braz J Nutr). 2017;30(6):713-22. | 3 |
| Silva JVL et al, 2010 | Rev bras epidemiol. 2010;13(1):83-93. | 3 |
| Skau JKH et al, 2014 | Am J Clin Nutr. 2014;99(1):130-38. | 3 |
| Skinner JD et al, 1999 | Pediatrics. 1999;103(1):58-64. | 3 |
| Skinner K et al., 2012 | Am J Health Promot. 2012;26(6):e159-70. | 4 and the study does not reach the inclusion criteria 1 |
| Slater A et al, 2010 | Public Health Nutr. 2010;13(8):1221-28. | The study does not reach the inclusion criteria 1 |
| Smith AEand Lloyd-Still JD, 1983 | J Pediatr. 1983 Nov;103(5):820-24. | 3 |
| Strucińska M et al, 2015 | Rocz Panstw Zakl Hig. 2015;66(4):353-60. | 3 |
| Suitor CW and Gleason PM, 2002 | J Am Diet Assoc. 2002;102(4):530-36. | 3 |
| Tambalis KD et al, 2018. | Journal of Clinical Sleep Medicine. 2018; 14(10): 1689-96 | 4 |
| Taylor C et al, 2013 | Perspect Public Health. 2013 Nov;133(6):330-36. | 4 e 7 |
| Thompson D et al, 2008 | Prev Med. 2008;47(5):494-97. | 4 |
| Thompson D et al, 2016. | Journal of the Academy of Nutrition and Dietetics. 2016; 116(9):1443-49 | 4 |
| Torok K et al, 2003 | Orv Hetil. 2003;144(6):259-62. | 10 |
| Trafalska E, 2014 | Rocz Panstw Zakl Hig. 2014;65(1):27-33. | 3 |
| Vereecken CA et al, 2005 | Eur J Clin Nutr. 2005;59(5):658-67. | The study does not reach the inclusion criteria 1 |
| Vereecken CA et al, 2008 | Int J Obes (Lond). 2008;32(5): S26-34. | The study does not reach the inclusion criteria 1 |
| Vereecken CA et al, 2013 | Public Health Nutr. 2013;16(1):15-26. | 4 |
| Verwied-Jorky S et al, 2011 | J Pediatr Gastroenterol Nutr. 2011;52(1):96-102. | 3 |
| Vieira TCL et al, 2007 | Arq Bras Cardiol. 2007;88(6):624-28. | 3 |
| Volger S et al, 2017 | Asia Pac J Clin Nutr. 2017;26(1):104-09. | 3 |
| Weker H et al, 2011 | Medycyna wieku rozwojowego. 2011;15(3):224-31. | 10 |
| Were GM et al, 2008 | J Food Agric Environ. 2008;6(2):68-73. | 3. |
| Williams CL et al, 2002 | J Am Coll Nutr. 2002;21(1):62-71. | 4 |
| Williamson DA et al, 2003 | J Am Diet Assoc. 2003;103(9):1139-45. | The study does not reach the inclusion criteria 1 |
| Williamson DA et al, 2004 | Eat Weight Disord. 2004;9(1): 24-28 | The study does not reach the inclusion criteria 1 |
| Wilson AM and Lewis RD, 2004 | J Am Diet Assoc. 2004;104(3):373-78. | 3 |
| Wiseman N, Harris N and Downes M, 2017 | Int J Behav Nutr Phys Act. 2017;14(1):1-9. | 4 |
| Ymamah GAN et al, 2018 | RJPBCS. 2018;9(4):542-46. | 3 |
| Yousefichaijan P et al, 2018 | Iran J Pediatr. 2018; 28(2):e9196. | 3 |
| Zachwieja J et al, 2001 | Pol Merkur Lekarski. 2001;10(58):237-40. | 10 |
